# Increasing growth rate slows adaptation when genotypes compete for diffusing resources

**DOI:** 10.1371/journal.pcbi.1007585

**Published:** 2020-01-07

**Authors:** Jeremy M. Chacón, Allison K. Shaw, William R. Harcombe

**Affiliations:** University of Minnesota, Department of Ecology, Evolution, and Behavior, St. Paul, Minnesota, United States of America; Wageningen University, NETHERLANDS

## Abstract

The rate at which a species responds to natural selection is a central predictor of the species’ ability to adapt to environmental change. It is well-known that spatially-structured environments slow the rate of adaptation due to increased intra-genotype competition. Here, we show that this effect magnifies over time as a species becomes better adapted and grows faster. Using a reaction-diffusion model, we demonstrate that growth rates are inextricably coupled with effective spatial scales, such that higher growth rates cause more localized competition. This has two effects: selection requires more generations for beneficial mutations to fix, and spatially-caused genetic drift increases. Together, these effects diminish the value of additional growth rate mutations in structured environments.

## Author summary

What determines how quickly a beneficial mutation will spread through a population? The intuitive answer is that mutations that confer faster growth rates will spread at a rate that is relative to the size of the growth-rate benefit. Indeed, this is true in a well-mixed environment where all genotypes compete globally. But most organisms don’t live in a simple well-mixed environment. Many organisms, like bacteria, live in a structured environment, such as on the surface of a solid substrate. Does life on a surface change the expectation about the spread of faster-growing mutants? We developed a mathematical model to answer this question, and found that on a surface, the actual growth rates—not just the relative growth rates—were critical to determining how fast a faster-growing mutant spread through a population. When the simulated organisms grew slowly, competition was basically global and a faster-growing mutant could pre-empt resources from far-away competitors. In contrast, when organisms grew more quickly, competition became much more localized, and the faster-growing mutant could only steal resources from neighboring competitors. This result means that there are diminishing returns to series of mutations which confer growth-rate benefits. This idea will help us predict and understand future and past evolutionary trajectories.

## Introduction

Species can adapt to environmental change through the fixation of beneficial alleles. If the rate of fixation is too low, species may face consequences such as extinction in changing environments [1,2]. Life in a spatially-structured habit is generally thought to slow the rate of adaptation [3–6]. This is because in spatially-structured environments, competitive interactions are more likely to be localized, and so individuals with a beneficial mutation compete more often with their own genotype than with ancestors [4]. However, even in the presence of spatial structure, slow resource acquisition rates can result in population dynamics which resemble a well-mixed system [7]. Therefore, it is uncertain whether the effect of spatial structure on the rate of adaptation is constant or dependent on the biological and environmental details of the species examined.

Whether rare genotypes are benefited or harmed by spatial structure is context-dependent. For example, a rare allelopathic genotype can benefit from spatial structure when it is surrounded by susceptible competitors [8,9]. However, this benefit of structure can become a detriment if a change in founder density puts allelopaths too far from susceptible targets or too close to cheaters [9,10]. When spatial structure increases the degree to which competition is just with neighbors (i.e. increases the competition localization), it can compound other mechanisms which affect the rate of adaptation. For example, epistatic interactions between mutations can result in reduced rates of adaptation that diminish the effect of additional mutations [11–14], and these diminishing returns can be compounded by spatial structure [2]. Similarly, spatial structure can increase genetic drift and founder effects, thereby reducing the rate of adaptation [7,15]. However, neither of these negative effects of spatial structure on the rate of adaptation are universal: they can be ameliorated by increased dispersal rates or resource diffusion, which serve to make a system more well-mixed [4,16,17]. Therefore, to predict the evolution of species in spatially-structured environments, it is imperative to quantitatively understand what variables results in an environment with high competition localization, and the causal consequences of such localization on the rate of adaptation.

Here we test how growth rate affects competition localization in a spatially-structured environment, and what the consequences of this are on the rate of adaptation. We hypothesized that, due to the localizing effect that high resource uptake has on competitive interactions [7], higher basal growth rates will increase the number of generations required for beneficial mutations to fix.

## Results

**Fig 1:**
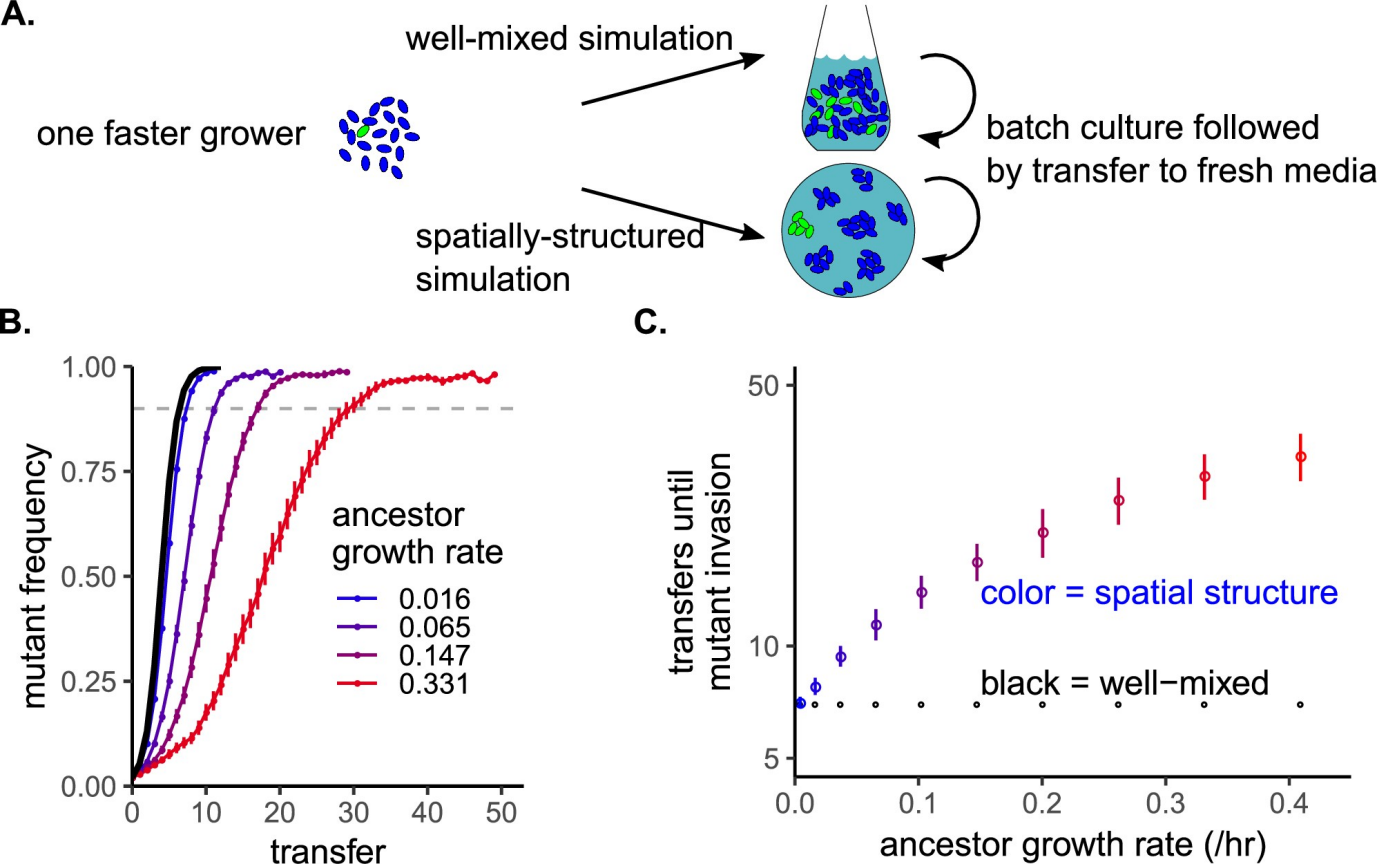
In populations with higher basal growth rates, more generations are required for faster-growing mutants to invade in a spatially-structured environment. A) Schematic for the simulation experiment using resource-explicit models with Monod growth. Within a transfer, cells grew until resources were consumed. Then, cells were deterministically diluted and transferred to fresh environment (with random locations in spatial simulations). This continued until mutant invasion. B) Time series of invasion of faster-growing mutants in simulations, plotted as the mutant frequency versus transfer number. The black line is the time series for all well-mixed simulations (they perfectly overlap regardless of ancestor growth rate). The other colors are for spatially-structured simulations with different ancestor growth rates. Error bars are standard error over twenty replicates. The faster-growing mutant always had a 10% growth rate advantage over its ancestor competitors. The dashed horizontal line indicates the cutoff frequency that was used to determine the number of transfers required for invasion. C) The number of transfers the mutant required to reach a frequency of 0.9, plotted versus ancestor growth rate. Colors correspond to the same simulations in B.

We used reaction-diffusion simulations to test how the absolute growth rate of a population influences the time required for invasion by a mutant with a 10% increase in growth rate. We examined growth rates from 0.01 to 0.4 / hr, which includes a range observed across multiple species in many environments as referenced in the Bionumbers database [18]. Simulations were run in environments that were either well-mixed or spatially-structured on a torus. Each transfer began with 49 founders. The starting transfer (transfer 0) began with one founder being the 10% faster-growing mutant. Each transfer’s simulation ran until 99% of the resources were consumed. Then, we applied a bottleneck to the population, selecting 49 new founder cells proportional to final genotype frequency at the end of the previous transfer to seed a fresh environment. Transfers continued until the faster-growing mutant genotype reached 90% frequency. In spatially-structured environments, founder cells were randomly arranged each transfer, with different sets of randomizations used for each replicate simulation. Resources were initially homogeneously distributed, and spread via diffusion as they were consumed (Fig 1A).

The ancestor’s growth rate had no effect on number of transfers required for the faster-growing mutant to invade a well-mixed environment (black line, Fig 1B and 1C). However, in a spatially-structured environment, increasing the ancestor’s growth rate increased the number of transfers required for invasion (Fig 1B and 1C). This relationship was robust to changes in the growth rate benefit, the half-saturation constant of resource use, the amount of resources available in the environment, and the number of founder cells (Supplementary S1 Fig). However, the pattern was not observed if resources were replenished as in a chemostat rather than with serial, “seasonal” pulses (Supplementary S1 Fig).

Why did increasing absolute growth rates increase the number of generations required for faster mutants to invade a seasonal spatially-structured habitat? Compared to well-mixed habitats, previous work has shown that the rate of adaptation is slower overall in spatially-structured habitats because interactions are more localized [3–6]. We therefore hypothesized that faster ancestor growth rates increased the degree of competition localization. We specifically define “competition localization” as the degree to which a colony competes with neighbors versus all possible competitors. We sought to test this hypothesis by quantifying competition localization as a function of ancestor growth rate during a single spatial simulation without transfers.

**Fig 2:**
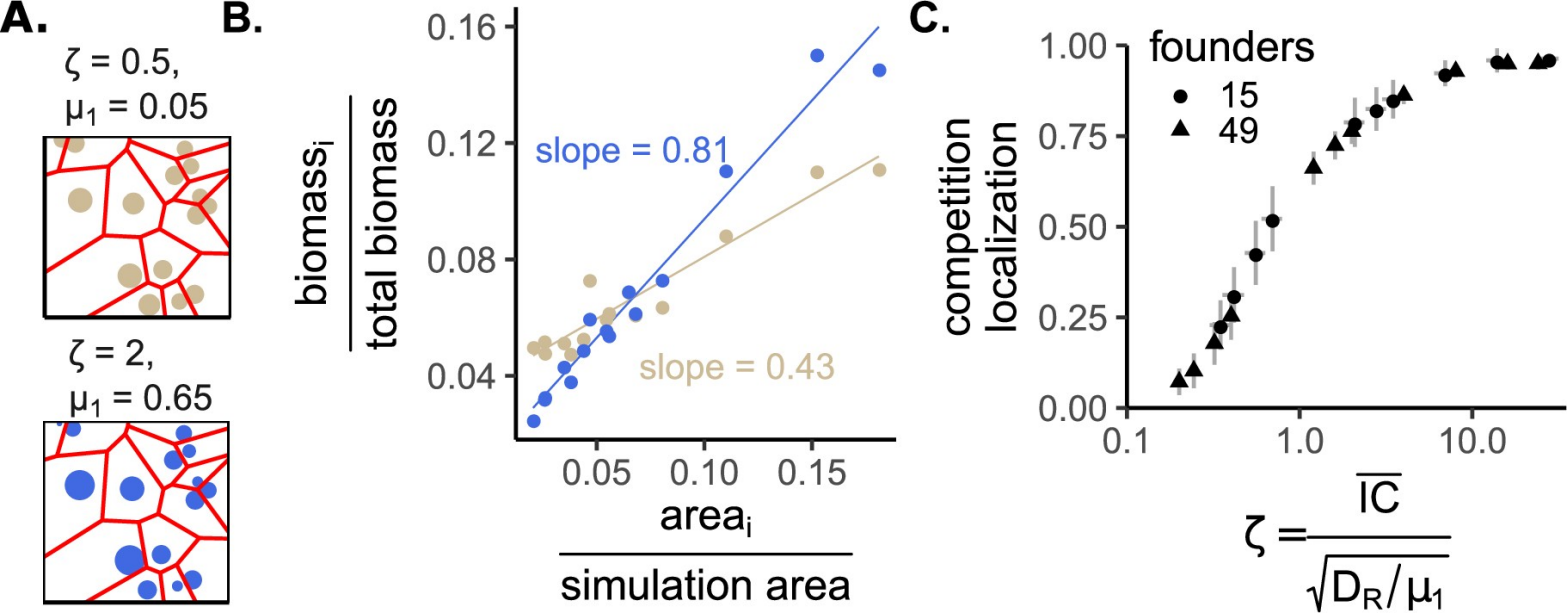
Competition localization increases with growth rate or the distances between colonies, and decreases with the resource diffusion constant. A) Two simulation “maps” with the same founder cell geometry but different growth rates. Circle size is proportional to final biomass of colonies begun with a single founder cell in a spatial environment after resources have been exhausted. The lines designate the Voronoi polygons. The text above each simulation diagram shows *ζ* on top. On the bottom we show the growth rate (μ_1_) back-calculated from the scaled model’s *ζ* when the resource diffusion constant is similar to that of glucose in agar and the simulation area is 2.5cm X 2.5cm. B) A plot of relative biomass (biomass of a focal colony divided by the summed biomass of all colonies) versus relative polygon area (polygon area of a focal colony divided by the total simulation area) for the two simulations shown in A. Each point represents a different colony. The lines are linear least-squares fits, and the slope of each line is our measurement of competition localization. C) Competition localization versus *ζ* for two different founder densities. Each point is the mean competition localization for a given growth rate and founder density. Vertical error bars are standard deviation of 10 replicates each with different founder locations. Horizontal error bars are standard deviation of *ζ* due to different mean nearest-neighbor distances across maps ($\bar{IC}$, see Materials and Methods).

To measure competition localization, we used an approach we previously showed can be used to understand how spatial territory size influences the final biomass of colonies [7]. First, the simulation area was converted into a Voronoi diagram (Fig 2A). This generates a polygon for each founder cell that encloses all of the simulation area that is closer to the focal founder than to any other founder. In other words, the boundaries (and therefore area) of a founder’s polygon are set by its neighbors. By plotting normalized final colony biomasses versus the colonies’ normalized polygon areas and fitting a line to this data, we can measure the degree to which colonies’ biomasses scale with their polygon areas (Fig 2B). A large slope close to one indicates that neighbors that dictate polygon boundaries are the most important competitors and therefore competition localization is high. Smaller slopes mean that neighbors have a smaller impact on colony biomass, and competition localization is low (i.e. competition is global). We previously verified this logic by showing that when the slope was close to one, repeating simulations where one founder cell was removed only affected the growth of colonies in neighboring Voronoi polygons [7]. Therefore, we deem this slope “competition localization,” and use it as a response variable to test whether competition localization is affected by growth rate.

We wanted to determine how physiological and environmental parameters interacted to influence competition localization. Applying dimensional analysis to our model revealed a natural length scale for the spatial dimensions that is coupled to growth rate and the diffusion constant of the resource (*x*_*c*_ and *y*_*c*_, see Materials and Methods, and [19] for a similar scale in a Lotka-Volterra model). In the model, founder cells occupy specific spatial positions, and we can measure the average of the minimum distances between colonies: $\bar{IC}$. Higher $\bar{IC}$ occur when founder cell density is lower. Interestingly, when $\bar{IC}$ is scaled to produce *ζ*, the *scaled* mean intercolony distance, we find that squared increases in growth rate (μ_1_), or squared decreases in the resource diffusion constant (*D*_*R*_), are equivalent to linear increases in the distances between competitors (see Methods for details; Supplementary S2 Fig for the results from Fig 1 viewed through this scaling; and Supplementary S3 Fig for calculations on simplified simulation maps):

$$\zeta =\frac{\bar{IC}}{\sqrt{D_{R}/\mu_{1}}}$$

Consistent with our initial results, increasing the natural length scale (which can be interpreted as increasing growth rate) led to more localized competition (Fig 2C, increasing x-axis values for a given founder number). Furthermore, *ζ* was able to simultaneously capture the effect of changes in founder density on localized competition (Fig 2C, different founder numbers).

**Fig 3:**
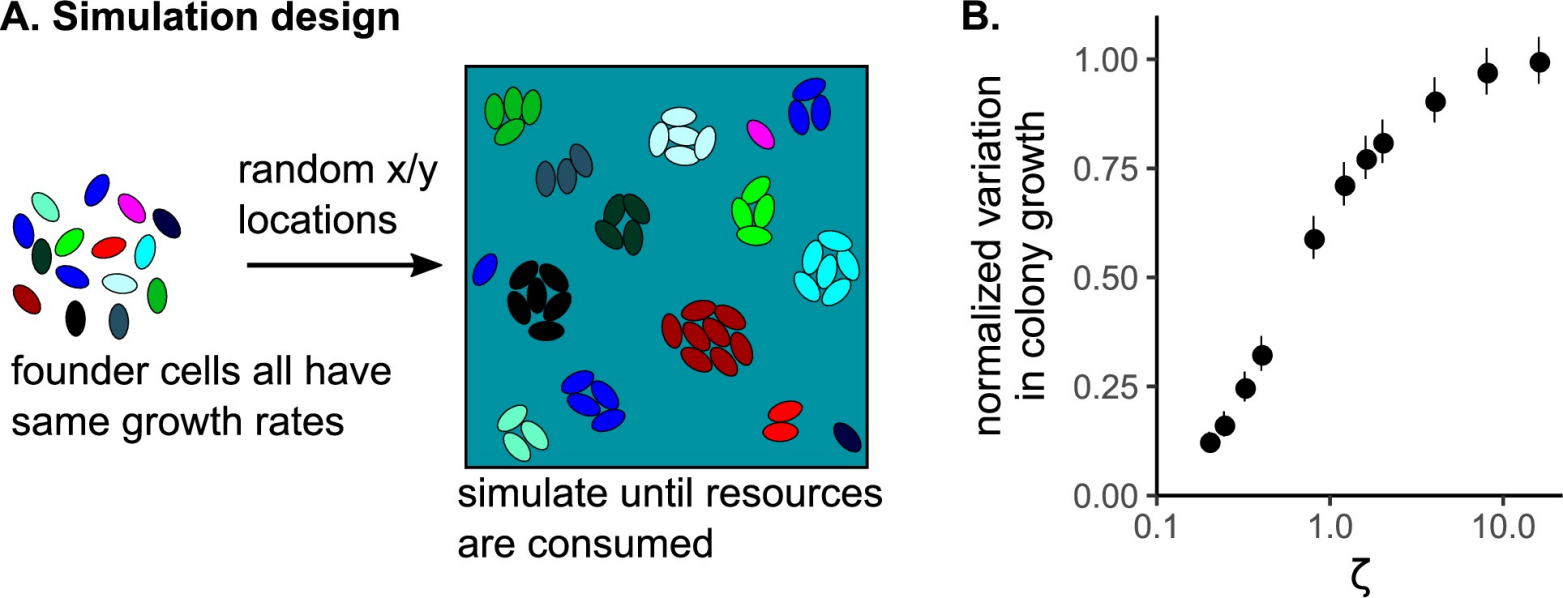
Increasing competition localization increases genetic drift. A) Experimental design for the simulations. B) The variation in frequency of the physiologically-identical genotypes, as a function of *ζ*. The points are means, averaged across replicates, of the standard deviation of the final genotype frequency. These means were normalized by dividing by the maximum standard deviation. The error bars are standard error. The horizontal error bars are standard error of the mean of *ζ*, due to different founder locations causing different $\bar{IC}$ (see Materials and Methods).

We next tested why increasing competition localization decreased the rate of adaptation. Genetic drift can be a result of interactions occurring more locally [15]. Therefore, we examined the effect of *ζ* on the rate of genetic drift among a set of genotypes which had the same growth rate and began at the same genotype frequency. We ran simulations with 49 founder cells. Fifteen different founder maps were used, with different random locations (and therefore different $\bar{IC}$). For each map, we tested multiple values of the natural length scale, equivalent to testing multiple values of growth rate (μ_1_). Since all founders began with the same growth rate, any change in genotype frequency must be due to genetic drift that arose as a result of the stochastic placement of founder cells on the surface. The stochastic nature of initial founder location caused different founders to have different Voronoi polygon areas, which may influence their growth and cause variability in final genotype frequency. We found that the variability in final genotype frequency increased when *ζ* increased (Fig 3).

**Fig 4:**
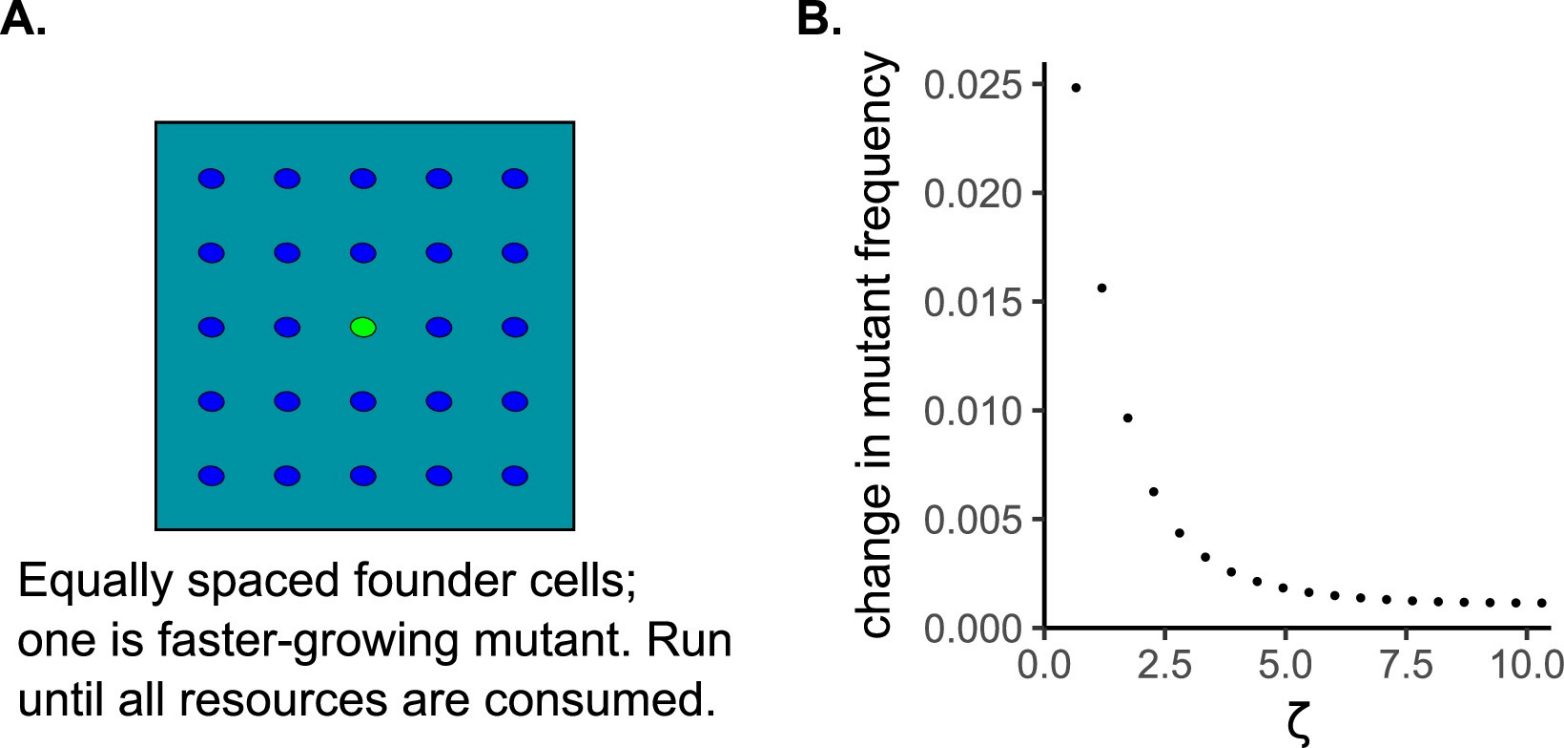
Selection decreases when growth rates are higher in a spatially-structured environment. A) Simulation design with a faster growing mutant (green) in the midst of evenly spaced ancestors. In these simulations, *ζ* increased only by increasing the natural length scale, which is equivalent to increasing growth rate (μ_1_). B) The absolute increase in frequency of the mutant genotype as a function of *ζ*.

Finally, we examined how competition localization might affect the rate of selection in an environment without genetic drift. We setup simulations in which founder cells were arranged in a grid with equal inter-colony spacing which removed the stochastic effects of variable Voronoi polygon areas. The ‘center’ cell was a mutant with a 10% growth rate advantage (Fig 4A). We ran simulations along a gradient of *ζ* by varying the natural length scale only (i.e. not also randomizing founder locations to vary $\bar{IC}$), and measured the increase in frequency of the mutant after resources were exhausted. The mutant had a smaller increase in frequency, and therefore was selected for less strongly, when *ζ* was larger (Fig 4B).

## Discussion

We showed that higher ancestral growth rates diminish the rate of invasion of a proportionally-faster-growing mutant. The reason for this is that higher basal growth rates cause cells in a spatially-structured environment to compete more locally. The increased competition localization slows selective adaptation generally, because an invading mutant can only preempt resources from neighbors and not more distant competitors. The increased competition localization also increases genetic drift because the size of one’s founding territory becomes a more important determinant of how many offspring a founder will have. Taken together, our results suggest that through evolutionary time increasing growth rate will have progressively diminishing fitness benefits, possibly leading to selection for phenotypes other than growth rate.

We found that in our model, growth rates were inextricably linked to the distances between competing colonies and the diffusion constant of the limiting resource. While we focused on the influence of growth rate, our model shows that we would obtain similar results by reducing the density of founders (thereby increasing inter-competitor distances) or by reducing the diffusion constant of the resource. This result helps explain why reducing nutrient diffusion promotes coexistence of a strong and weak competitor [20]. Interestingly, our simulations showed that sometime competition in spatially-structured habitats is global: when growth rates were low enough, spatial position did not alter the outcome of any given founder cell. We expect that continued work which treats growth rates, inter-competitor distances, and resource diffusion rates as interacting parts of one ecosystem property (interaction localization) will help shed light on the sometimes confusing effects of spatial structure.

Our main result on the role of growth rate in reducing the rate of adaptation was robust to many changes in the model: the degree of growth-rate improvement of the invading mutant, the half-saturation resource consumption constant, and the total number of founding cells. However, the result did *not* occur in a chemostat-like environment. Chemostats and batch cultures differ in other ways as well. For instance, chemostats generally select for species which can grow at the lowest resource concentrations [21], whereas batch culture environments select for high maximum growth rates [22,23]. Wild environments likely exist in a continuum from seasonal batch culture to pure chemostat, and yet most research focuses on one extreme or the other. We hypothesize that studying model communities in environments that blend aspects of chemostats and batch cultures—as well as better delineating the modes of resource replenishment and mortality in natural systems—will significantly improve our understanding of eco-evolutionary dynamics.

Our simulations used a single limiting resource, with a fixed diffusion constant, in a homogeneous environment. We expect that determining a general version of ζ in more complex situations, for example where species experience co-limitation by multiple resources each with different diffusion constants, will be non-trivial. Nevertheless, we hypothesize that the qualitative result showing that higher growth rates slow adaptation will hold true, as long as the mutant and its ancestors occupy the same niche. However, it is less clear what the outcome will be when colonies alter the diffusion constant of their limiting resource directly, as occurs when resources transition from diffusion in agar to diffusion into the heights of a colony, or when colonies secrete surfactants [24,25]. These ecosystem engineering events may change how local the interactions are between colonies, with important implications for the direction and rate of evolution.

Decreasing rates of adaptation to a specific environment are commonly observed (e.g. [26]). The bio-physical cause of diminishing returns shown here is distinct from the diminishing returns due to genetic effects described in well-mixed populations. In well-mixed populations, adaptation can slow because epistatic interactions reduce the benefit of secondary mutations [11,12,27]. Here, we showed that diminishing returns result from the fact that increases in growth rate serve to strengthen the effect of spatial structure. High growth rates make it more likely a founder population competes only with neighbors, which decreases the proportion of environment-wide resources the founder consumes, and therefore decreases the founder’s change in allele frequency. In well-mixed populations only relative growth rates matter, while in structured environments absolute growth rates also play a critical role in determining the fate of alleles.

Higher ancestral growth rates reduce selection and are therefore likely to influence evolution of other phenomena. Toxin producers generally gain an advantage from high density because they get more of a return for killing competitors [9]. Interestingly, the optimal toxin production rate is predicted to decrease with increases in density because too much production increases the chance that cheaters reap the returns from killed competitors [10]. Our results suggest that this relationship will change depending on absolute growth rates, because higher growth rates tend to counteract the effects of higher density. Relatedly, since high growth rates are more likely to restrict interactions to neighbors, we would expect that fast-growing cooperator species are more easily able to exclude cheaters [28].

Considered broadly, our results suggest that absolute growth rate alters the evolutionary trajectory of sessile organisms that compete for diffusing resources. While we modeled bacteria in a homogenous environment, we expect a similar effect in scenarios such as when plants compete for nutrients or water in the soil. Higher growth rates will generally reduce the local pool of resources, thereby reducing selection and increasing genetic drift. The inextricable relationship between growth rate, density and localization of interactions alters fundamental properties of evolutionary trajectories.

## Materials and methods

### Model development

All the simulations used a reaction-diffusion partial-differential equations model. Bacterial genotypes (*B*_*i*_ where i is genotype i out of n different genotypes (usually, n = 2)) grew based upon their maximum growth rate (*μ*_*i*_), the local resource concentration (R), and Monod kinetics with a half-saturation constant (k). As bacteria grew, resources were consumed with an efficiency parameter (λ). Both bacteria and resources spread via diffusion, with separate diffusion constants (*D*_*B*_ and *D*_*R*_). Parameter units and explanations are in Table 1. The following equations are the “full” model:

$$\frac{\partial B_{i}}{\partial t}=diffusion\;in\;x\;and\;y\;directions+growth=D_{B}(\frac{\partial^{2}B_{i}}{\partial x^{2}}+\frac{\partial^{2}B_{i}}{\partial y^{2}})+B_{i}\mu_{i}\frac{R}{R+k}$$

$$\frac{\partial R}{\partial t}=diffusion\;in\;x\;and\;y\;directions-consumption=D_{R}(\frac{\partial^{2}R}{\partial x^{2}}+\frac{\partial^{2}R}{\partial y^{2}})‐\sum_{i=1}^{n}{\lambda B}_{i}\mu_{i}\frac{R}{R+k}$$

**Table 1:** Description of model variables and parameters.

**Unscaled variable or parameter**	**Units**	**Definition or interpretation**	**Typical value used at t = 0**
*B*_*i*_	cells	Amount of cells of bacterial genotype i	49, distributed randomly on lattice
*R*	resources	Amount of resources	100 per lattice box
*D*_*B*_	cm^2^/h	Diffusion constant of the bacterial cells	1.8e-5cm^2^/h (= 5e-9 cm^2^/s)
*μ*_*i*_	1/h	Maximum per-capita growth rate of bacterial genotype i	Multiple, from 0.01–0.4
*k*	resources	Amount of resources in local environment at which point the bacterial cells’ per-capita growth rate is half its maximum per-capita growth rate	1
*D*_*R*_	cm^2^/h	Diffusion constant of the resource	1.8e-2cm^2^/h (= 5e-6 cm^2^/s)
*λ*	Resources / cells	Yield coefficient; the amount of resources required for growth of one cell	1
**Scaled variable or parameter**	**Scaling procedure**	**Interpretation of scaled, dimensionless, variable or parameter**	**Typical value used at t = 0**
$\hat{R}$	*R*/*k*	Loosely, the amount of resources. More accurately, a dimensionless local resource abundance that when > 1 confers bacterial growth that is faster than half-maximum.	100 per lattice box
$\hat{B_{i}}$	*B*_*i*_ *λ*/*k*	The amount of bacterial cells.	49, distributed randomly on lattice
*D*_*c*_	*D*_*B*_/*D*_*R*_	Relative diffusion constant of bacteria versus resource	0.001
*μ*_*c*_	*μ*_2_/*μ*_1_	In invasion assays, there were two genotypes, *μ*_1_ (the faster-growing mutant) and *μ*_2_ (the slower-growing ancestors). *μ*_*c*_ is the relative maximum growth rate of slower-growing ancestor versus faster-growing mutant	1/1.1

We rescaled this model. First, we made state variables dimensionless by division with characteristic quantities of the same dimension:

$$\hat{R}=\frac{R}{R_{c}},\;\hat{B_{i}}=\frac{B_{i}}{B_{c}},\;\hat{t}=\frac{t}{t_{c}},\;\hat{x}=\frac{x}{x_{c}},\;\hat{y}=\frac{y}{y_{c}}$$

The characteristic variables are defined as:

$$R_{c}=k,\;B_{c}=\frac{k}{\lambda },\;t_{c}=\frac{1}{\mu_{1}},\;x_{c}=y_{c}=\sqrt{t_{c}D_{R}}=\sqrt{\frac{D_{R}}{\mu_{1}}}$$

We redefine the growth rate to be relative to the growth rate of the first genotype (which, if genotypes have different growth rates, is always the fastest-growing):

$$\mu_{i,rel}=\mu_{i}/\mu_{1}$$

And we redefine the diffusion constant of the bacteria to be proportional to the resource diffusion constant:

$$D_{B}=D_{R}D_{c}$$

With the chain rule and some algebra this simplifies the reaction-diffusion model into the following set of equations, which we refer to as the “scaled” model (hats are omitted for clarity):

$$\frac{\partial B_{i}}{\partial t}=D_{c}(\frac{\partial^{2}B_{i}}{\partial x^{2}}+\frac{\partial^{2}B_{i}}{\partial y^{2}})+B_{i}\mu_{i,rel}\frac{R}{R+1}$$

$$\frac{\partial R}{\partial t}=(\frac{\partial^{2}R}{\partial x^{2}}+\frac{\partial^{2}R}{\partial y^{2}})‐\sum_{i=1}^{n}B_{i}\mu_{i,rel}\frac{R}{R+1}$$

The most interesting result of this scaling, discussed in the Results, is that the variables representing space ($\hat{x}$ and $\hat{y}$) are a function of ‘real’ space (x and y) as well as bacterial growth rates and resource diffusion:

$$\hat{x}=\frac{x}{\sqrt{D_{R}/\mu_{1}}},\hat{y}=\frac{y}{\sqrt{D_{R}/\mu_{1}}}$$

In multiple simulations, we examined outcomes that varied as a function of the average minimum intercolony distance scaled by growth rate and the resource diffusion constant: ζ. ζ was calculated in two steps. First, we computed a population-level spatial summary statistic, $\bar{IC}.\;\bar{IC}$ is the arithmetic mean of the minimum center-to-center intercolony distance, *IC*_*i*_, in centimeters, for each colony *i* in the simulation. Second, we applied the same scaling we did to get $\hat{x}$ to $\bar{IC}:\;\zeta =\bar{IC}/\sqrt{D_{R}/{ゼ}_{1}}$, and is unitless. As described in the results, changing growth rate (μ_1_) changes ζ, as does altering the density or positions of founder cells ($\bar{IC}$).

### Simulation details and analysis

The simulations shown in Fig 1 used the full model. For these simulations, a toroidal world with 5 cm per “side” was simulated, discretized into 101 x 101 boxes (i.e. ~0.05cm / box). D_*R*_ = 5e-6 cm^2^ / s, which is typical for a small sugar in water or a 1% agar Petri dish [7]. D_*B*_ = 5e-9 cm^2^ / s, i.e. 1000x smaller than the constant for the resource. Each unit of resource could be converted into one cell, making λ =1. k = 1, meaning when there was one cell equivalent of resource left, the growth rate was at half-maximum. We also tested k = 50 (Supp. S1A Fig). At the beginning of each transfer, each lattice box contained 100 units of resource. We also tested 10,000 and 1,000,000 units of resource per box (Supp. S1C Fig). *μ*_1_ (the maximum growth rate of the mutant) was always equal to 1.1 *μ*_2_, where *μ*_2_ was the maximum growth rate of the ancestor. Bacteria were seeded at 49 randomized locations with one unit of biomass per location. We also tested 25 and 98 founders (Supp. S1B Fig). In the first simulation, one of the founders was the faster-growing mutant. With these boundary conditions, the simulation was run until >99% of all resources were consumed. Once this threshold was reached, the frequency of the faster-growing mutant was calculated. A new environment was generated with new founder locations, and the starting frequency of the bacteria was, allowing for rounding, equal to the final frequency from the previous simulation. These batch-transfer simulations continued until the mutant reached 90% frequency. Twenty replicate batch-transfer experiments were simulated per growth rate, with different founder bacterial locations in each replicate. We also performed equivalent simulations (same founder densities, resource concentrations, growth rates) in mass-action liquid environments.

Simulations testing the relationship between ζ and competition localization (Fig 2) used the scaled model. Each simulation environment used a lattice with 101 x 101 boxes. We used two different founder densities: 15 or 49 cells. For each founder density treatment, we generated 10 different founder ‘maps,’ in which the founder locations were placed in different random locations (maximum 1 founder / location). Each map / founder density combination had a different $\bar{IC}$, and the $\bar{IC}$ differed more between founder numbers than between founder maps within a founder number. For each map, we varied growth rate (μ_1_) over eleven values. Note that since these simulations used the scaled model, μ_1_ is not an explicit parameter. In the simulations, μ_1_ was varied by varying the natural length scale $\sqrt{D_{R}/{ゼ}_{1}}$, which is equivalent to varying μ_1_ when the resource diffusion constant (*D*_*R*_) is held constant. In total there were 220 simulations for Fig 2: 11 μ_1_ X 2 founder densities X 10 founder maps. To track the growth from each founder cell, each founder had its own differential equation (and therefore *μ*_*i*,*rel*_ was equal to 1 for all founders within a map, since they were equivalent). The diffusion constant of the bacteria was 1/1000 that of the resource, in dimensionless units. At the beginning of the simulation each lattice box contained 100 units of resource. These simulations used a square (rather than toroidal) lattice, to simplify the spatial analysis. Simulations were run until > 99% of the resources were consumed, i.e. the bacteria in all simulations went through the same number of generations. In Fig 2, each point’s x-axis value is the average of ζ from the 10 simulations with a given growth rate (μ_1_) and founder number, with the small amount of variation caused by different founder maps shown with the horizontal standard deviation error bars.

Once simulations were complete, competition localization was measured. This was done by measuring the Voronoi polygon area for each founder cell using the dirichletAreas function of the spatstat package [29] in R. Polygon areas were scaled into relative polygon areas by dividing each area by the total simulation area. Relative final biomasses were calculated by dividing the total biomass from each founder cell by the total biomass in the simulation. The competition localization was then calculated by finding the slope of the linear regression of the relative biomass versus the relative polygon areas (Fig 2B).

Simulations testing the relationship between ζ and the rate of genetic drift (specifically, variance in the frequency of neutral genotypes with equivalent growth rates, Fig 3) used the same simulation results as the simulations testing the relationship between ζ and the Voronoi response.

Simulations examining the influence of ζ on selection when genetic drift cannot have any effect (Fig 4) used the scaled model with a toroidal lattice of 105 x 105 boxes, each holding 100 units of resource. 49 founder cells were placed at equidistant locations in a grid. One founder had a 10% growth rate advantage (*μ*_1,*rel*_ = 1.1). Simulations were run until >99% of the resources were consumed, and the change in frequency of the growth-rate mutant was measured.

All simulations were coded and run in R [30]. While we initially planned on using the ReacTran package [31] to numerically solve each reaction-diffusion simulation, we found that we could run the simulations much faster and with smaller errors if we iteratively performed each growth step (done per-box using deSolve’s ode function [31]) and diffusion step (using all boxes). Diffusion calculations used a simple forward finite differences scheme, and therefore the time step was kept < = 0.1 * dx^2^ / D, where dx was the width of a box and D was the maximum diffusion constant. This ensured accuracy of the diffusion results. R simulations were run using the University of Minnesota’s Minnesota Supercomputing Institute. Code to run example simulations is provided in the supplementary file ‘S1 Code’.

## Supporting information

S1 Code Contains two files of R code which demonstrate how to run simulations as in the manuscript. (ZIP)

S1 Fig The influence of ancestor growth rate on mutant invasion in a spatially-structured, seasonal environment is robust but does not occur in a chemostat-like environment. A) The number of transfers the mutant required to reach a frequency of 0.9, plotted versus ancestor growth rate. This is similar to Fig 1C, but includes the results from simulations with either a lower benefit or a higher k. The blue data are the same as from Fig 1C. B) Like Fig 1C, but showing the results from simulations with different founder numbers. The simulation size was constant across these treatments, so a higher founder number implies a higher founder density. The blue data are the same as from Fig 1C. C) Like Fig 1C, but showing the results from simulations with higher resource concentrations (and therefore productivity). The data in blue are the same as from Fig 1C, which used simulations with a concentration of 100 resources per box. The red and green data are from simulations with 100-fold and 10,000-fold more resources per box, respectively. The circles are the results in spatial simulations, and the triangles are the results in well-mixed simulations. D) The number of hours until a 10% faster-growing mutant genotype reached 90% frequency in a spatial chemostat simulations. The vertical line indicates the chemostat dilution rate (0.1 / hr). Simulations with growth rates below this did not survive and are not plotted. These simulations were similar to those in Fig 1. A 105x105 box lattice was used to simulate. Boxes each began with 100 resource units. Forty-nine cells were randomly arranged on the lattice. One of these cells was a 10% faster-growing mutant. These simulations different from the main text simulations in that resources were replenished, in each box, from a reservoir with 100 resource units. Additionally, both resources and cells were diluted through time. The dilution rate, which governed replenishment and dilution, was 0.1 / hr. No transfers were performed. Variation in time arose because of the initial random founder placement. Error bars are standard error of 20 replicates.
(TIF)

S2 FigThe role of the diffusion constant or *ζ* on the time required for a faster-growing mutant to invade.The equation for *ζ* allowed us to consider the results from Fig 1 as a function of the resource diffusion constant or, more generally, as a function of *ζ*, rather than as a function of growth rate. A) The number of transfers until the faster-growing mutant reached 90% frequency plotted versus the resource diffusion constant. For this plot, we took the results from Fig 1C and calculated *ζ* when $\bar{IC}$ = 0.45cm (here, since different starting reps and different transfers all had different maps, $\bar{IC}$ is calculated under the simplifying assumption that founder cells are arranged in a grid). Then, to consider the results in terms of the diffusion constant (*D*_*R*_), we fixed the ancestor growth rate at 0.15 /hr and solved the *ζ* equation for *D*_*R*_, which is plotted on the x-axis. B) The number of transfers until the faster-growing mutant reached 90% frequency plotted versus *ζ*.(TIF)

S3 FigDiagram showing calculation of *ζ* for three different founder maps and two growth rates.A) Three different simplified simulation lattices are shown. Note that map 3 has twice the density of maps 1 and 2. To the right of each map shows the calculation of the mean nearest neighbor distance ($\bar{IC}$), when dx (the lattice box width) = 0.1cm. B) A table showing calculations of *ζ* for the three different maps in A. For these calculations, a resource diffusion constant of 5e-6 cm^2^ / s was used. The top half shows calculations when dx = 0.1cm, the bottom half shows calculations when dx = 0.2cm (which causes a doubling of $\bar{IC}$). There are calculations for two different growth rates (μ_1_ = 0.1 or 0.4). The shaded parts of the table draw attention to the fact that multiplying $\bar{IC}$ by some factor (here, 2) has the same effect on *ζ* as does multiplying μ_1_ by the square of that factor (here, 2^2^).(TIF)
