# Supplementary figures and images for: Increasing growth rate slows adaptation when genotypes compete for diffusing resources
Source: PLoS Comput Biol. 2020 Jan 7;16(1):e1007585. doi: 10.1371/journal.pcbi.1007585 (PMC6946136; doi:10.1371/journal.pcbi.1007585)

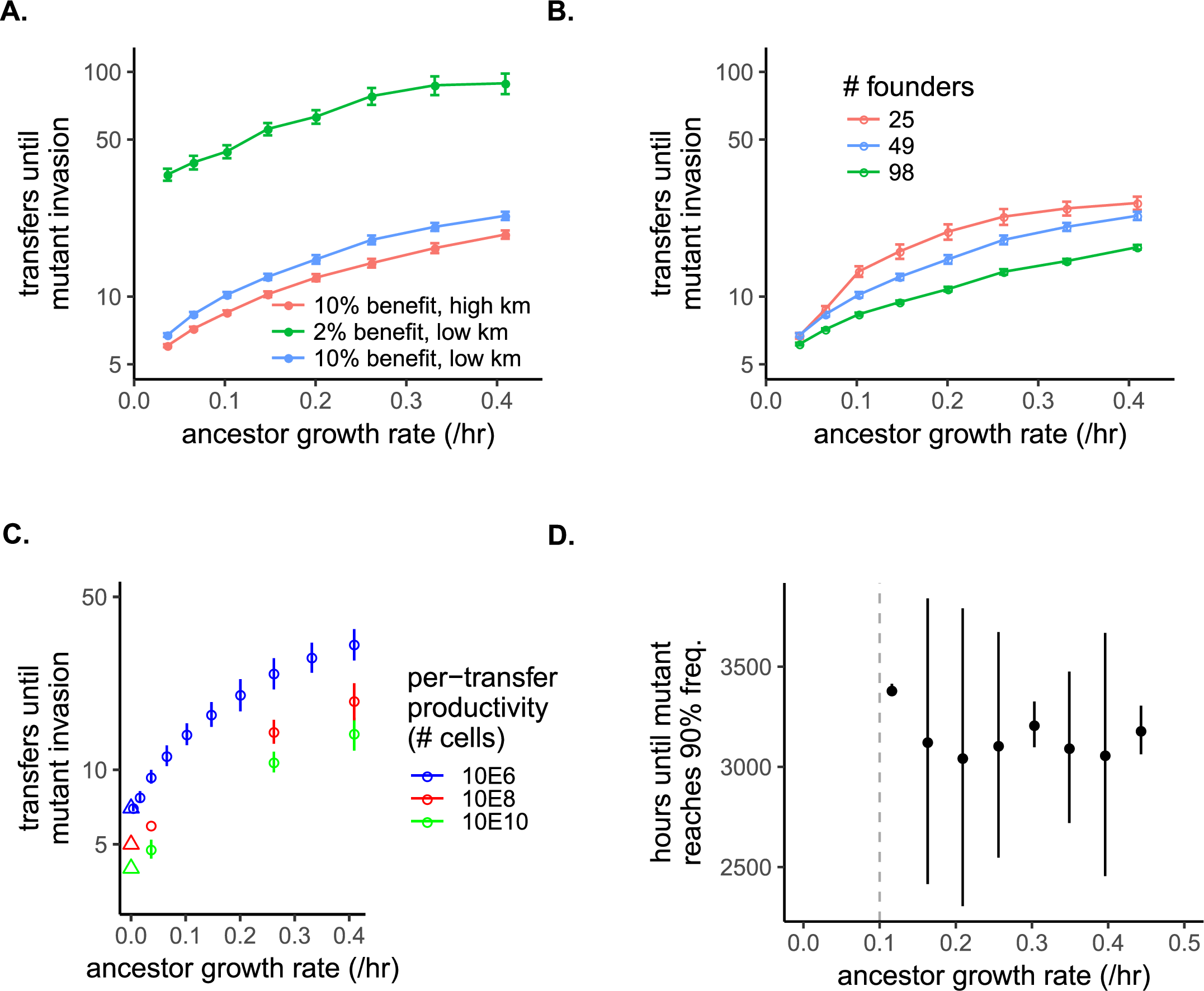

Supplement: S1 Fig — A) The number of transfers the mutant required to reach a frequency of 0.9, plotted versus ancestor growth rate. This is similar to Fig 1C, but includes the results from simulations with either a lower benefit or a higher k. The blue data are the same as from Fig 1C. B) Like Fig 1C, but showing the results from simulations with different founder numbers. The simulation size was constant across these treatments, so a higher founder number implies a higher founder density. The blue data are the same as from Fig 1C. C) Like Fig 1C, but showing the results from simulations with higher resource concentrations (and therefore productivity). The data in blue are the same as from Fig 1C, which used simulations with a concentration of 100 resources per box. The red and green data are from simulations with 100-fold and 10,000-fold more resources per box, respectively. The circles are the results in spatial simulations, and the triangles are the results in well-mixed simulations. D) The number of hours until a 10% faster-growing mutant genotype reached 90% frequency in a spatial chemostat simulations. The vertical line indicates the chemostat dilution rate (0.1 / hr). Simulations with growth rates below this did not survive and are not plotted. These simulations were similar to those in Fig 1. A 105x105 box lattice was used to simulate. Boxes each began with 100 resource units. Forty-nine cells were randomly arranged on the lattice. One of these cells was a 10% faster-growing mutant. These simulations different from the main text simulations in that resources were replenished, in each box, from a reservoir with 100 resource units. Additionally, both resources and cells were diluted through time. The dilution rate, which governed replenishment and dilution, was 0.1 / hr. No transfers were performed. Variation in time arose because of the initial random founder placement. Error bars are standard error of 20 replicates. (TIF) [file pcbi.1007585.s002.tif]

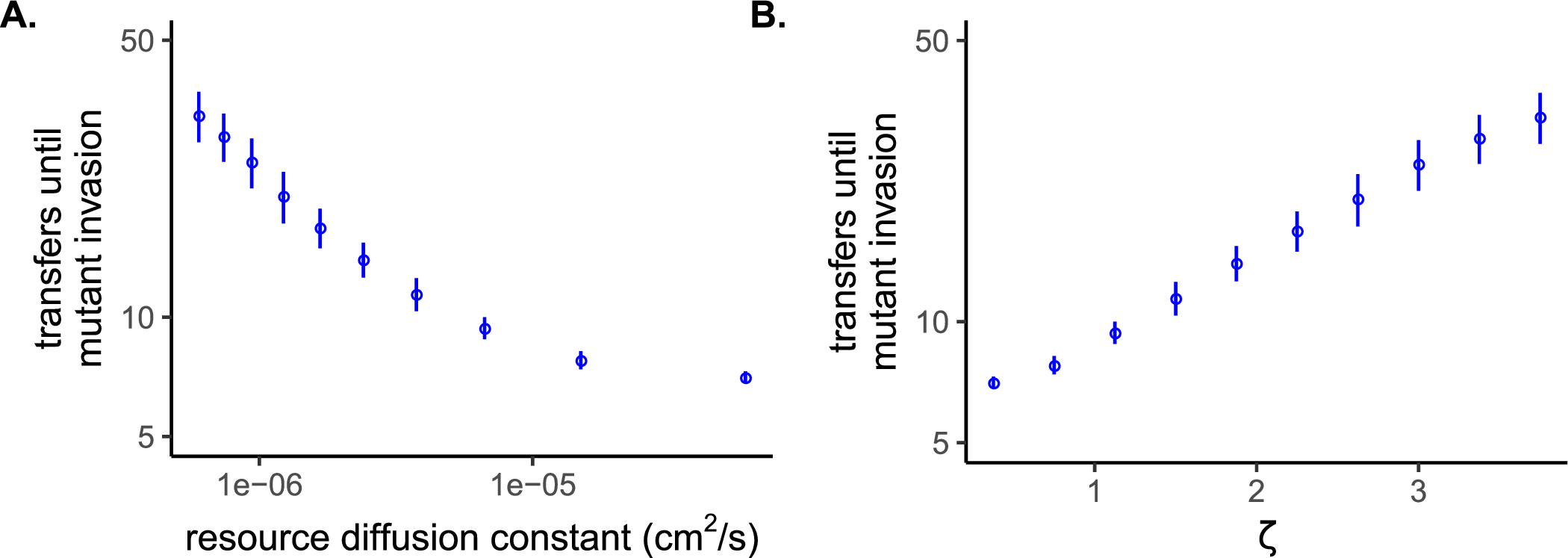

Supplement: S2 Fig — The equation for ζ allowed us to consider the results from Fig 1 as a function of the resource diffusion constant or, more generally, as a function of ζ, rather than as a function of growth rate. A) The number of transfers until the faster-growing mutant reached 90% frequency plotted versus the resource diffusion constant. For this plot, we took the results from Fig 1C and calculated ζ when IC¯ = 0.45cm (here, since different starting reps and different transfers all had different maps, IC¯ is calculated under the simplifying assumption that founder cells are arranged in a grid). Then, to consider the results in terms of the diffusion constant (DR), we fixed the ancestor growth rate at 0.15 /hr and solved the ζ equation for DR, which is plotted on the x-axis. B) The number of transfers until the faster-growing mutant reached 90% frequency plotted versus ζ. (TIF) [file pcbi.1007585.s003.tif]

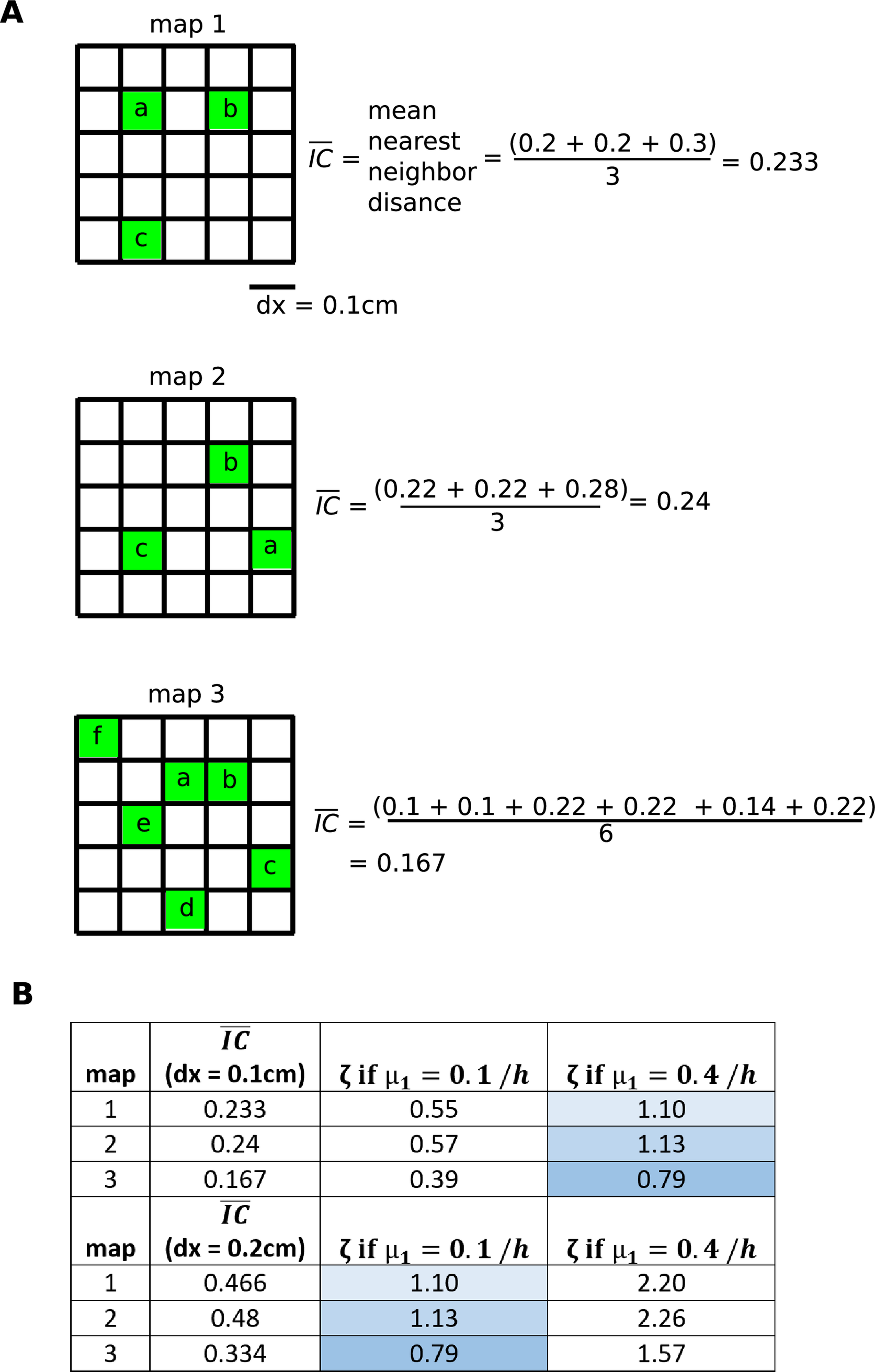

Supplement: S3 Fig — A) Three different simplified simulation lattices are shown. Note that map 3 has twice the density of maps 1 and 2. To the right of each map shows the calculation of the mean nearest neighbor distance (IC¯), when dx (the lattice box width) = 0.1cm. B) A table showing calculations of ζ for the three different maps in A. For these calculations, a resource diffusion constant of 5e-6 cm2 / s was used. The top half shows calculations when dx = 0.1cm, the bottom half shows calculations when dx = 0.2cm (which causes a doubling of IC¯). There are calculations for two different growth rates (μ1 = 0.1 or 0.4). The shaded parts of the table draw attention to the fact that multiplying IC¯ by some factor (here, 2) has the same effect on ζ as does multiplying μ1 by the square of that factor (here, 22). (TIF) [file pcbi.1007585.s004.tif]
